# The primary spine practitioner as a new role in healthcare systems in North America

**DOI:** 10.1186/s12998-022-00414-8

**Published:** 2022-02-09

**Authors:** Donald R. Murphy, Brian Justice, Christopher G. Bise, Michael Timko, Joel M. Stevans, Michael J. Schneider

**Affiliations:** 1 Department of Family Medicine, Alpert Medical School of Brown University, 133 Dellwood Road, Cranston, RI 02920 USA; 2 Excellus BlueCross BlueShield, 165 Court Street, Rochester, NY 14647 USA; 3 Department of Physical Therapy, University of Pittsburgh, Bridgeside Point 1, 100 Technology Drive, Suite 210, Pittsburgh, PA 15219-3130 USA; 4 Department of Physical Therapy, University of Pittsburgh, Bridgeside Point Suite 228, 100 Technology Drive, Suite 210, Pittsburgh, PA 15219-3130 USA; 5 Department of Physical Therapy, University of Pittsburgh, Bridgeside Point 1, 100 Technology Drive, Suite 500, Pittsburgh, PA 15219-3130 USA

**Keywords:** Low back pain, Neck pain, Health care reform, Primary care, Health policy, Spine, Physical therapy, Physiotherapy, Chiropractic, Implementation science

## Abstract

**Background:**

In an article published in 2011, we discussed the need for a new role in health care systems, referred to as the Primary Spine Practitioner (PSP). The PSP model was proposed to help bring order to the chaotic nature of spine care. Over the past decade, several efforts have applied the concepts presented in that article. The purpose of the present article is to discuss the ongoing need for the PSP role in health care systems, present persistent barriers, report several examples of the model in action, and propose future strategies.

**Main body:**

The management of spine related disorders, defined here as various disorders related to the spine that produce axial pain, radiculopathy and other related symptoms, has received significant international attention due to the high costs and relatively poor outcomes in spine care. The PSP model seeks to bring increased efficiency, effectiveness and value. The barriers to the implementation of this model have been significant, and responses to these barriers are discussed. Several examples of PSP integration are presented, including clinic systems in primary care and hospital environments, underserved areas around the world and a program designed to reduce surgical waiting lists. Future strategies are proposed for overcoming the continuing barriers to PSP implementation in health care systems more broadly.

**Conclusion:**

Significant progress has been made toward integrating the PSP role into health care systems over the past 10 years. However, much work remains. This requires substantial effort on the part of those involved in the development and implementation of the PSP model, in addition to support from various stakeholders who will benefit from the proposed improvements in spine care.

## Introduction

Over 25 years ago Waddell described the management of low back pain as a “twentieth century medical disaster” [1]. This was followed by a series of publications calling for comprehensive changes in the approach to low back pain (LBP) by health care systems [2–7]. Despite all this discussion, the situation in the twenty-first century has actually worsened. Spine-related disorders (SRDs), defined here as various disorders related to the spine that produce axial pain, radiculopathy and other related symptoms, are now the most prevalent cause of disability and lost workdays around the world [8, 9], and spending has skyrocketed [10].

A recent strategy has been to increasingly shift focus in healthcare toward primary care. In nearly all areas of medicine, emphasizing primary care practitioners as the initial point of contact for most patients leads to decreased reliance on specialists [11]. However, in the area of SRDs this strategy is problematic. Medical education has not provided primary care practitioners with an extensive background in musculoskeletal diagnosis and management, including evidence-based nonpharmacologic approaches [12, 13].

A recent pragmatic trial of patients with acute LBP treated in primary care found that almost half of the patients received recommendations that were not consistent with leading LBP guidelines [14]. More alarming was the finding that exposure to guideline-non-concordant primary care was an independent risk factor for the transition from acute to chronic LBP. Attempts to provide education about LBP guidelines to improve primary care for patients with SRDs have largely failed [15]. In the area of SRDs, a different approach is needed.

In 2011, a commentary article was published entitled “The establishment of a primary spine care practitioner and its benefits to health care reform in the United States” [16]. This article was an expansion of a concept initially introduced by Haldeman [17], who advocated for the use of practitioners specifically trained in primary-level spine care. Others have since written on this topic [18].

We define the primary spine practitioner (PSP) as *a specially-trained clinician (most often a chiropractor or physical therapist) who practices at the top of their license, playing a primary role in the diagnosis, management, referral, and case coordination of patients with SRDs*. The model proposes that this specialized professional is needed in the primary-level management of patients with SRDs. The knowledge, skills and responsibilities required of this professional are detailed in our initial paper [16].

The PSP model responds to the need for greater efficiency and effectiveness in the management of patients with SRDs [6]. There has been increased attention to this issue in recent years. However, a predominantly low-value environment still dominates spine care, in which medication, imaging, injection and surgery are emphasized [19]. Significant inroads into the PSP model have taken place over the past 10 years, although health care systems as a whole have not yet fully embraced the concept. The purpose of this article is to discuss the evolution and present status of the PSP model, the role of the PSP in the larger picture of “primary spine care”, and future strategies toward broader adoption of the PSP role.

## Primary spine care and the PSP

“Primary Spine Care” is a general term that refers to any activity involving a primary-level health care professional who has early contact or may participate in the “front end” care and coordination of a patient with a SRD [20]. These activities include diagnosis, investigation, management, patient education, and referral to other healthcare providers. We use the term “Primary Spine Practitioner” to identify one of the key professionals who participate in primary spine care.

We propose that the ideal application of the PSP role is to serve as the first contact for majority of patients with SRDs, analogous to the role that the general dentist plays in the area of oral health [16]. However, we recognize that many of these patients will continue in the foreseeable future to consult with primary care practitioners, emergency departments, and urgent care personnel as their first-contact providers for spine care. Therefore, in the context of “Primary Spine Care” we suggest that another useful application of PSPs is to function as intermediary providers between traditional primary care practitioners and specialists. This application of primary spine care requires the coordinated activity of “front end” practitioners that include generalists (e.g., traditional primary care practitioners) and focused primary-level practitioners (PSPs). We posit that the vast majority of patients with SRDs can be managed purely at the primary spine care level, without the need for diagnostic investigations, specialty referrals or invasive procedures.

The primary spine care model has the potential to provide many benefits to different stakeholders in health care systems (although more data are needed, and thus some of these potential benefits are speculative), including:

- *Patients*: A more coordinated, consistent, efficient and affordable management of SRDs. This may lead to better clinical outcomes and greater patient satisfaction [21].
- *Providers*: Reduced burden on primary care practitioners [21], and for specialists a shorter wait time and a more appropriate case mix [22].
- *Employers*: Reduced direct and indirect costs associated with SRDs, such as earlier return to work, increased productivity and decreased absenteeism.
- *Payers*: Reduced variation in the management of SRDs, and lower costs per case as a result of improved decision making and a decrease in inappropriate imaging and invasive procedures [23].
- *Governments*: More efficient utilization of the healthcare workforce by enabling physical therapists (PTs) and chiropractors (DCs) to work at the “top of their license” [24], easing the burden on other medical personnel.

## Barriers to the implementation of the PSP model

Several barriers were identified in our previous publication [16]. Many of these barriers still remain today, but significant strides have been made in several areas.

### Educational changes

Although there has not been widespread adoption of PSP training in North America, we have encountered less resistance than we had 10 years ago [16]. In fact, some institutions have moved toward preparing their students to play the PSP role [25, 26].

In addition, the University of Pittsburgh has developed a post-professional PSP certificate program, which was initially launched in December of 2017 [27]. This program consists of combined online distance education and live weekend workshops, which have been organized into four “Units” of instruction. The four units total about 120 h of instruction over the course of approximately one year.

Applicants are PTs and DCs who are required to have certain prerequisite skills such as differential diagnosis/ medical screening, manipulation and other manual therapies, and application of rehabilitative exercise. Attendees are taught advanced application of evidence-informed, patient-centered spine care as well as team-based care and functioning in the role of PSP, providing “primary care for spine patients”. Learning objectives include: primary diagnosis and management, self-care management, application of the principles of Cognitive-Behavioral Therapy [28], Acceptance and Commitment Therapy [29] and Motivational Interviewing [30], decision-making regarding referrals and follow-up, disability management, inter-professional communication, and more.

### Legislative and regulatory changes

A number of proposals have arisen in recent years to improve the efficiency and effectiveness of health care. The Institute for Health Care Improvement has identified the need for practitioners to work “at the top of their license” in maximizing utilization of available professionals [24]. The Institute of Medicine has called for a “retooling” of the existing workforce [31], to enable professionals to play new roles and to bring needed innovations, designed to improve value in health care. Dower et al. [32] have emphasized the need to restructure professionals’ scopes of practice to empower them to fill new roles. Goertz et al. recognized the need of such “redeployment” as it specifically applies to the PSP model [18]. However, significant legislative and regulatory obstacles exist that impair the ability of non-allopathic professionals, particularly DCs and PTs, to fully engage in the provision of PSP services.

For example, in many jurisdictions neither DCs nor PTs have the right to order appropriate imaging and other diagnostic tests, or to refer patients to other professionals when necessary. Even in those jurisdictions in which DCs have diagnostic rights, financial obstacles are in place, such as the Medicare policy in the US that requires patients to pay out-of-pocket for imaging and other special tests when ordered by a DC [33]. While in some areas legislation has enabled patients to directly access a PT without referral, often referred to as “direct access”, this is often undermined by payer policies that do not cover PT services without referral from a medical doctor.

Modernization of legal statutes, practice acts and payer policies is needed to keep up with necessary changes in the delivery of value-based spine care, and to allow professionals to function “at the top of their license” [24].

### Incentivizing value in spine care

The Accountable Care Organization movement in the US, which is designed to “manage the full continuum of care and be accountable for the overall costs and quality of care for a defined population” [34], has grown considerably. This has provided channels for the novel implementation of primary spine care services [21, 23]. Further, greater calls have been made both within and outside the US for more efficient and cost-effective primary-level spine care [5, 6, 35, 36].

But significant barriers remain. First, it is common to encounter a “top-heavy” environment in spine care in which systems are incentivized to focus on high-cost and often low-value services [37]. Second, the relatively low—or completely absent—reimbursements made by payers to DCs and PTs, along with high co-payments, create significant barriers to the full implementation of PSP services. Health care systems in a fee-for-service environment typically derive substantially greater income from high-cost, invasive procedures than from low-cost, minimally invasive services, regardless of the benefit to individuals and to society.

Third, specific barriers to accessing DCs and PTs commonly exist [38]. For example, Medicare in the US provides coverage for only one service provided by a DC, i.e., spinal manipulation [33]. The financial responsibility falls on the patient for all other services necessary in providing primary spine care, such as physical examinations and supervised exercise. In addition, the care provided by a PT or DC often requires a series of sessions, the cost of which is partially or completely charged to the patient. Therefore, the total cost to the patient for visits to these practitioners can be substantially greater than the cost to see an interventionalist or surgeon. All of these barriers ironically create an incentive for patients to pursue care that is often inappropriate and of low-value [38]. Some efforts to remedy this situation have recently been instituted [39] but many more are needed.

### Overcoming professional prejudice

A common assumption is that it is always necessary for patients with SRDs to first be seen by a medical doctor [40]. This assumption is not supported by evidence; when a patient with LBP sees a DC or PT as the first contact provider, greater value is realized in terms of clinical benefit, decreased cost to the system [41–43] and decreased opioid use [44]. There has been a shift in patient and professional attitudes toward acceptance of PTs and DCs as front-end managers of SRDs [45–47], but there is room for further progress. The PSP model seeks to expand beyond mere high-quality treatment to the application of primary care for patients with SRDs [16]. There is an opportunity to bring about open-mindedness regarding the role non-allopathic professionals (PSPs) can play in an evolving model of primary spine care.

### Implementation and sustainability

There are two key barriers to implementation and sustainability of the PSP model. First, the majority of “spine centers” still focus on the use of secondary-level spine specialists, as opposed to primary-level practitioners, at the front end of care [48]. Second, many hospitals and medical groups are invested in a low-value environment that rewards them for providing more care, not necessarily better care [37]. These barriers inhibit health systems from hiring PTs and DCs to serve in the PSP role in providing high-value spine care.

However, increased demand for ‘thinking outside the box” is seen from multiple stakeholders involved in—or affected by—spine care, which has facilitated a greater appreciation for new and innovative approaches to SRDs [36]. This has led to several innovative ways in which the PSP model has been implemented, including in primary care [49, 50], hospital systems [21, 51], a payer system [23] and underserved communities [52]. Table 1 lists several of these successful applications of primary spine care in different clinical environments.

**Table 1:** Examples of environments in which the primary spine practitioner model is in place. This is not necessarily a complete list

Location	Program	Year Started	Environment	Direct access or referral?
Plymouth, Massachusetts, USA [21]	Spine Care & Neurosurgery Center	2009	Hospital-based	Both
New York State [23]	Excellus Spine Health Program	2014	Payer system community	Both
Botswana, India, Ghana, Dominican Republic [52]	World Spine Care	2008	Free standing clinics	Direct access
Eastern Massachusetts, USA	Atrius Health Integrated Spine Program	2015	Multi-specialty healthcare system	Both
Hanover, New Hampshire, USA [49, 50]	Primary Spine Care	2017	Primary care clinic	Both
Ontario, Canada [22]	Rapid Access Clinics	2012	Community	Referral
Pittsburgh, Pennsylvania, USA	University of Pittsburgh Medical Center Program for Spine Health	2018	Community	Referral
Chicago, USA	Midwest Orthopaedics at Rush	2018	Hospital-based	Both
Fargo, ND USA	Sanford Health Spine Center	2018	Community	Both

The Global Spine Care Initiative [7] and the Lancet series “Low Back Pain” [6, 53, 54] proposed a series of recommendations that respond to many of the obstacles to implementation. They laid out the problems in spine care [6, 55], and introduced world-wide initiatives designed to bring about greater efficiency and effectiveness, and to reduce the tremendous global burden of SRDs [7]. Among the several recommended solutions is a widespread focus on the application of primary care in the management of patients with SRDs, by professionals with *appropriate knowledge and skill* [35, 53]. This has brought significant attention to the need, not only for improved spine care at all levels, but specifically for effective, evidence-based *primary* spine care.

## Future strategies for broader implementation of the PSP model

We envision broader implementation of the PSP model arising from several efforts, including:

- Expanding PSP training from its relatively localized focus in the northeast of the US to other regions of North America and internationally, including an expanded use of online distance learning [27].
- Shifting post-professional PSP training from didactic and workshop orientation to a clinical-based residency format.
- Expanding the implementation of spine care pathways [56, 57] rooted in primary spine care, with the PSP playing a key role.
- Entry-level professional programs, particularly in physical therapy and chiropractic medicine, placing greater focus on evidence-based spine care that prepares students for the PSP role.
- Engaging and educating government agencies on the need for scope of practice expansions that allow the PSP to practice as an autonomous practitioner.
- Promoting recognition, trust and acceptance among the general public regarding the PSP role of PTs and DCs serving as a first-contact professionals for SRDs.
- Gathering further data on the impact of the primary spine care model in general, and the PSP role in particular, on patient outcomes, health care costs, system efficiencies and the “Quadruple Aim” [58]. One example of this is the recently-funded project by Goertz et al. [59].
- Responding to the chaotic “Spine Supermarket” [60] environment by educating the public regarding self-directed decision-making in taking charge of their own spine health.
- Expanding primary care practitioners’ awareness of the availability of PSPs, and the administrative and clinical benefits that arise from having a PSP in place on the primary care team [20, 49, 50].
- Advocating for expanded changes in reimbursement designed to incentivize patients to pursue high-value primary spine care services [39].
- Championing the role of PSPs as part of an effort to respond to the opioid crisis [61] by shifting emphasis away from pharmacologic to nonpharmacologic approaches.
- Supporting and promoting the world-wide expansion of primary spine care in underserved populations through World Spine Care [52].

## Conclusion

There is a need for innovative responses and solutions to the current inefficient, low-value situation in spine care. The PSP model is one such response. This emerging role in health care systems requires a cadre of professionals with the requisite knowledge and skill to bring greater efficiency, effectiveness and value to the management of patients with SRDs. In addition, it is essential for health care systems to be receptive to—and supportive of—this new innovative role.

The PSP model is growing, and has seen significant progress in development and implementation. However significant challenges remain. Further progress requires concerted and coordinated effort to accelerate the current pace. This necessitates stakeholder engagement, including the support of health care systems, payer groups, regulatory bodies and governmental agencies, and the continued participation of professionals involved in existing PSP environments. Improved care of patients, at decreased cost to society, is a genuine possibility, despite the recent problematic trend of disability and costs in spine care. The PSP role is a small but significant step in that direction.

## Data Availability

Not applicable.

## Abbreviations

PSP: Primary spine practitioner
SRDs: Spine related disorders
US: United States
PT: Physical therapist or physiotherapist
DC: Chiropractor
